# Aortic malignancy masquerading as infrarenal aortitis: A diagnostic challenge

**DOI:** 10.1016/j.jvscit.2026.102186

**Published:** 2026-02-13

**Authors:** Davina Daudu, Hunter Gurevic, Lakshmi Pothukuchi, Victoria van Winden, Anwar Choudhary

**Affiliations:** a Department of Vascular Surgery, Fiona Stanley Hospital, Murdoch, Western Australia, Australia; b Pathwest, Fiona Stanley Hospital, Murdoch, Western Australia, Australia

**Keywords:** Aortitis, Endovascular aneurysm repair, Retroperitoneal neoplasms, Sarcoma

## Abstract

Primary retroperitoneal aortic sarcoma is an exceptionally rare malignancy and may closely mimic inflammatory or infectious aortitis on clinical assessment and imaging. An 89-year-old man presented with abdominal pain, weight loss, and periaortic inflammatory change on computed tomography, raising concern for aortitis with possible impending rupture. Urgent endovascular aortic repair was performed. Despite antimicrobial and immunosuppressive therapy, symptoms and radiographic findings progressed. Subsequent computed tomography-guided biopsy demonstrated a high-grade retroperitoneal aortic sarcoma. The presence of the stent graft influenced perceived procedural safety and allowed tissue diagnosis that had initially been deferred. This case highlights the diagnostic challenge posed by malignant aortic disease presenting as presumed aortitis and underscores the importance of maintaining diagnostic vigilance in atypical cases.

Primary retroperitoneal sarcomas are rare tumors with a poor prognosis, comprising less than 1% of all adult cancers and often presenting with nonspecific features.^1^^,^^2^ When such tumors arise from the abdominal aorta, they are even more exceptional, with very few cases reported in the literature.^1^^,^^2^ Their insidious growth and anatomical/clinical/radiographic overlap with other more common vascular pathologies such as aneurysmal disease, aortitis, and acute aortic syndrome, which can result in diagnostic uncertainty.

This poses a diagnostic dilemma for vascular specialists involved in the care of these patients, where initial management decisions may be initially guided by the assumption of infectious or inflammatory aortitis.

We present the case of an elderly man initially investigated and empirically treated for a paraaortic mass presumed to be aortitis but was ultimately found to be an aortic malignancy. The patient provided consent for publication of the case details and images.

This case highlights the challenge of differentiating malignant from inflammatory and infectious aortic disease and underscores the importance of maintaining diagnostic vigilance when confronted with an atypical clinical presentation.

## Case report

**Fig 1:**
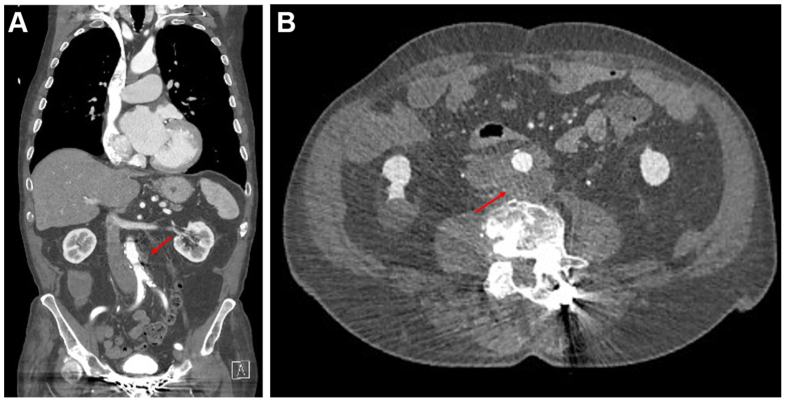
Computed tomography aortogram (CTA) on initial presentation, with *red arrows* pointing to the periaortic mass. **(A)** Coronal. **(B)** Axial.

An 89-year-old man was referred to our institution after undergoing a computed tomography (CT) with contrast, requested by the patient’s general practitioner for a 6-week history of lower abdominal pain. This pain was constant, with occasional sharp exacerbations that radiated to his back. He also had reduced appetite, 4 kilograms of weight loss in 6 weeks, and reduced frequency of bowel motions. He was hemodynamically stable, his abdomen was soft but tender in the right iliac fossa, and he had no signs of neurovascular compromise of his lower limbs. His white cell count was 8 × 10^9^/L, and his C-reactive protein was 22 mg/L. His CT angiogram (CTA) demonstrated inflammatory stranding and a low attenuation para-aortic mass posterior to the aorta at the level of L4, measuring 51 × 47 mm (anteroposterior × transverse), without aneurysmal degeneration (Fig 1).

His medical history included atrial fibrillation, chronic cardiac failure, ischemic heart disease, and a prior transient ischemic attack. He had no known personal or family history of aneurysmal disease. His surgical history included a previous bowel resection for diverticulitis and multiple previous lumbar laminectomies. He mobilized with no walking aids and was independent in activities of daily living. He was a nonsmoker, with minimal alcohol consumption.

## Initial admission

Given ongoing severe pain and progressive periaortic inflammatory change, concern was raised for impending rupture in the setting of presumed aortitis. An infrarenal endovascular stent graft was implanted within 24 hours of admission. The main body was a JOTEC E-tegra 26 mm × 80 mm × 110 mm, with a 16 mm × 6 mm left limb and 19 mm × 8 mm right limb. To reinforce the bifurcation, a 9 mm × 37 mm × 75 cm BeGraft was placed on the right and a 9 mm × 59 mm × 120 cm V12 on the left. The procedure was performed via bilateral percutaneous femoral artery access with pre-closure using ProGlide devices. Cannulation of the contralateral gate was not technically challenging.

### Infectious diseases and rheumatology teams were consulted

Investigations for infectious aortitis included bacterial and fungal blood cultures and serology for HIV, hepatitis B and C, *Coxiella burnetii*, *Burkholderia pseudomallei*, syphilis, and Q fever polymerase chain reaction with phase I and II IgG and IgM assays. Q fever phase II IgM serology was obtained as part of an infectious aortitis workup, given its recognized association with chronic vascular infection. Although the assay was positive, there was no relevant exposure history and confirmatory serology was negative, and this result was therefore interpreted by the Infectious Diseases team as a false positive. Autoimmune and vasculitic investigations were unremarkable. Erythrocyte sedimentation rate was 16 mm/hr.

Empiric intravenous amoxicillin–clavulanic acid was commenced with minimal biochemical response and only modest symptomatic improvement. CT-guided biopsy was offered but initially declined. The patient was discharged on oral antibiotics with outpatient follow-up.

## Second admission

**Fig 2:**
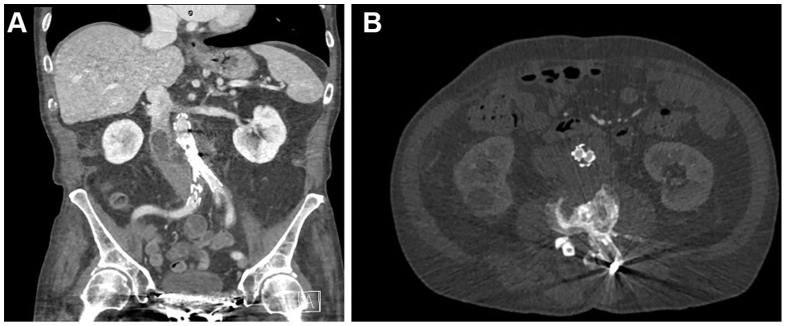
Computed tomography aortogram (CTA) on re-presentation. **(A)** Coronal demonstrating inferior vena cava (IVC) thrombus. **(B)** Axial.

He re-presented 2 weeks later with worsening right iliac fossa pain, with repeat CTA (Fig 2) demonstrating increased infrarenal aortic stranding, increased size of the paraaortic mass to 65 mm × 49 mm (anteroposterior × transverse), and thrombus within in the inferior vena cava. There was no lower limb edema, and no pulmonary embolism was detected. A caval filter was not placed. Intravenous piperacillin/tazobactam and oral anticoagulation were commenced.

**Fig 3:**
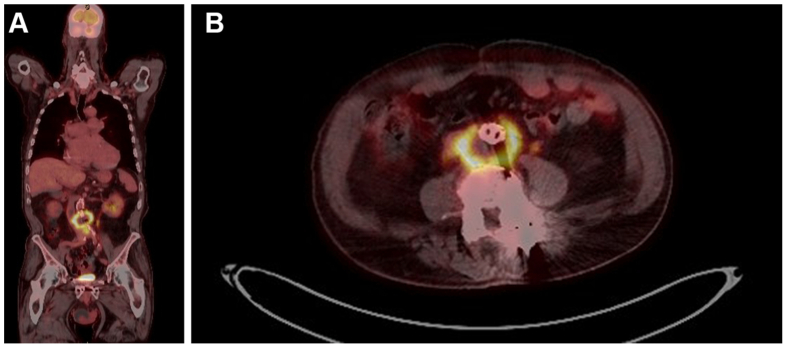
Positron emission tomography (PET) scan. **(A)** Coronal. **(B)** Axial.

Positron emission tomography (PET) imaging (Fig 3) demonstrated a diffusely PET-avid infrarenal aorta, thought to favor inflammatory aortitis. There was also mild avidity noted at the left hip intertrochanteric region. Magnetic resonance imaging of the left hip was performed, demonstrating cortical lesion in the left proximal femur of unclear etiology with no radiographically aggressive features. There was no evidence of lymphatic avidity.

Due to his ongoing pain and the PET scan findings suggestive of an inflammatory aortitis, he was commenced on 50 mg of oral prednisolone. He experienced significant symptomatic improvement within 24 hours of commencing steroids and no hemodynamic deterioration.

In the context of progressive mass enlargement, failure of antimicrobial therapy, and ongoing diagnostic uncertainty, CT-guided biopsy was pursued. The presence of the stent graft reduced perceived hemorrhagic risk and influenced procedural decision-making. The biopsy was performed without complication.

**Fig 4:**
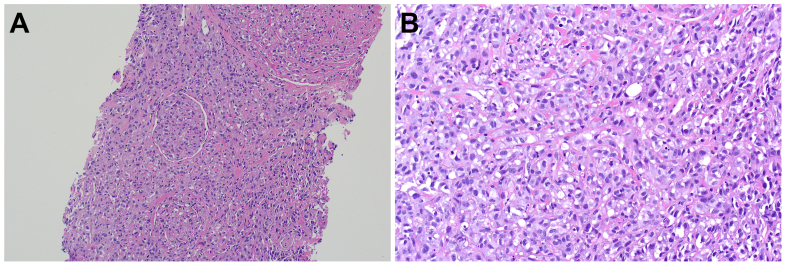
Histological slides. **(A)** Nested arrangement of malignant epithelioid cells within a hyalinized stromal background, with a focus of lymphovascular space invasion seen centrally. **(B)** High-power view of the malignant epithelioid cells demonstrating the nested architecture, nuclear pleomorphism, apoptosis, and mitotic activity.

The biopsy demonstrated a poorly differentiated/undifferentiated epithelioid malignancy surrounding a blood vessel (Fig 4). The tumor was composed of atypical epithelioid cells arranged in nests and cords with a rich capillary network throughout. Mitotic activity and tumor necrosis were seen. Lymphovascular space invasion was identified. Various carcinoma, melanoma, hematolymphoid, and mesenchymal immunohistochemical markers were performed, with no specific line of differentiation identified. The lesional cells only expressed smooth muscle actin, inhibin, and GATA3. Fluorescent in-situ hybridization testing was also performed for *MDM2* amplification, with no increase in copy number identified.

His case was discussed at the regional sarcoma multidisciplinary meeting and deemed unresectable and not suitable for palliative chemotherapy. The patient opted to pursue palliative care measures in the community and died within 2 months of hospital discharge.

## Discussion

Retroperitoneal sarcomas involving the abdominal aorta are very uncommon, with fewer than 300 documented cases described in the literature.^2^ Their rarity, combined with overlapping radiologic features, makes diagnosis challenging. Periaortic stranding and periaortic soft tissue thickening raise concern for infectious or inflammatory pathology, and in most instances a diagnosis of malignancy is only established intraoperatively or on histopathological analysis.^2^^,^^3^

Endovascular repair has been reported in selected cases of aortic malignancy. Thoracic endovascular aneurysm repair (EVAR) has been utilized in patients with thoracic aortic invasion to provide protection and allow subsequent biopsy.^4^ Similarly, endovascular strategies have been employed in cases of arch and descending aortic sarcoma presenting with embolic complications, where stent grafting facilitated further diagnostic and therapeutic steps.^5^ Other authors have described endovascular biopsy approaches for descending aortic masses.^6^ Additionally, there have been reports of angiosarcoma development after prior EVAR.[6], [7], [8] However, there is no established role for stent grafting in the management of presumed aortitis in the absence of aneurysmal degeneration or rupture, and endovascular intervention in this setting may complicate subsequent diagnostic evaluation.

In the present case, EVAR was performed urgently due to concern for impending rupture in the setting of persistent pain and progressive periaortic inflammatory change. In retrospect, this intervention would not be considered appropriate treatment for aortitis. Importantly, EVAR was not performed with diagnostic intent. Rather, the presence of the stent graft altered subsequent clinical decision-making and reduced perceived hemorrhagic risk, leading to biopsy that ultimately established the diagnosis.

## Conclusions

This case underscores the importance of considering malignant aortic disease when aortitis presents with atypical features such as focal mass-like morphology, progressive constitutional symptoms, or failure to respond to antimicrobial or immunosuppressive therapy. Early tissue diagnosis should be considered when diagnostic uncertainty persists.

## Funding

None.

## Disclosures

None.
